# The Oligostilbene Gnetin H Is a Novel Glycolysis Inhibitor That Regulates Thioredoxin Interacting Protein Expression and Synergizes with OXPHOS Inhibitor in Cancer Cells

**DOI:** 10.3390/ijms24097741

**Published:** 2023-04-23

**Authors:** Shivendra Singh, Flavia De Carlo, Mohamed A. Ibrahim, Patrice Penfornis, Alan J. Mouton, Siddharth K. Tripathi, Ameeta K. Agarwal, Linda Eastham, David S. Pasco, Premalatha Balachandran, Pier Paolo Claudio

**Affiliations:** 1 National Center for Natural Products Research, Research Institute of Pharmaceutical Sciences, School of Pharmacy, University of Mississippi, University, MS 38677, USA; 2 Cancer Center & Research Institute, Department of Pharmacology & Toxicology, School of Medicine, University of Mississippi Medical Center, Jackson, MS 39216, USA; 3 Department of Physiology, School of Medicine, University of Mississippi Medical Center, Jackson, MS 39216, USA; 4 Department of Biomolecular Sciences, School of Pharmacy, University of Mississippi, University, MS 38677, USA

**Keywords:** glycolysis, gnetin H, natural products, oligostilbene, Warburg effect

## Abstract

Since aerobic glycolysis was first observed in tumors almost a century ago by Otto Warburg, the field of cancer cell metabolism has sparked the interest of scientists around the world as it might offer new avenues of treatment for malignant cells. Our current study claims the discovery of gnetin H (GH) as a novel glycolysis inhibitor that can decrease metabolic activity and lactic acid synthesis and displays a strong cytostatic effect in melanoma and glioblastoma cells. Compared to most of the other glycolysis inhibitors used in combination with the complex-1 mitochondrial inhibitor phenformin (Phen), GH more potently inhibited cell growth. RNA-Seq with the T98G glioblastoma cell line treated with GH showed more than an 80-fold reduction in thioredoxin interacting protein (TXNIP) expression, indicating that GH has a direct effect on regulating a key gene involved in the homeostasis of cellular glucose. GH in combination with phenformin also substantially enhances the levels of p-AMPK, a marker of metabolic catastrophe. These findings suggest that the concurrent use of the glycolytic inhibitor GH with a complex-1 mitochondrial inhibitor could be used as a powerful tool for inducing metabolic catastrophe in cancer cells and reducing their growth.

## 1. Introduction

Cancer is one of the leading causes of death in humans, and its related morbidity and mortality constitute a significant health problem worldwide. Cytotoxic chemotherapy is still the main approach used in clinics, despite the tremendous efforts made to find novel treatments. Patients treated with chemotherapy often suffer from many side effects, such as hematological toxicity, cardiotoxicity, hepatotoxicity, and renal toxicity [1,2,3,4], and for these reasons, there is a great need to develop novel anticancer agents.

Natural products have been one of the most important and fundamental sources in drug discovery and development [5]. For decades, humans have relied on natural products for the prevention and cure of several diseases, and particularly in the case of anticancer drugs, more than 50% were discovered from natural products [6]. Additionally, several studies have demonstrated the potential of natural products in reducing cancer chemotherapy-associated side effects and/or increasing the therapeutic index of chemotherapeutic agents [7].

In an effort to discover novel anticancer therapeutics, more than 900 pure natural compounds isolated at the National Center for Natural Product Research of the University of Mississippi repository were screened through a battery of cancer-related signaling pathway assays to identify compounds exhibiting unique patterns of anticancer activities [8,9]. Among the compounds screened, we identified a new, unique activity/mechanism for gnetin H (GH), a resveratrol trimer, and an electrophilic compound that has been previously described by different research groups to inhibit the growth of several cancer cell lines [10,11,12,13,14,15]. This generated the rationale to investigate further the antineoplastic properties and mechanism of action of this compound and its synergistic action with complex-1 inhibitors.

The continuous evolution of cell biology research has increased our understanding of cancer as a complex disease. However, a number of common traits that support tumor formation, growth, and metastasis have been described by Hanahan and Weinberg in 2000 [16], often referred to as hallmarks of cancer. Particularly, it has become evident that tumor initiation and progression strongly rely on the reprogramming of cell metabolism, mainly to support increased needs for ATP generation and macromolecules biosynthesis, as well as to maintain tight control of redox balance. They suggested that most cancers, during their progression, acquire specific functional capabilities that provide growth and survival advantages to cancer cells and enable metastatic dissemination. Over the last two decades, reprogramming of energy metabolism to support neoplastic proliferation has been proposed as an emerging hallmark of cancer [17]. Metabolic changes associated with cancer have been described in detail by Pavlova and Thompson [18]. Enhanced glucose uptake, aerobic glycolysis, and lactate fermentation have been revealed as key features of many cancers [19]. Unlike normal differentiated cells that derive ATP from oxidative phosphorylation (OXPHOS), it has been well documented that most rapidly developing tumors depend primarily on glycolysis even when oxygen is available. This phenomenon is referred to as the “Warburg effect”, and it was originally hypothesized that dysfunctional mitochondria were the cause of such a metabolic phenotype [20,21]. It has been reported that cancer cells, driven by oncogenic mutations, heavily rely on glycolysis and the pentose phosphate pathway (PPP) for their high demand of energy, biosynthetic intermediates, and reducing equivalents, such as NAPDH [21,22,23]. These oncogenic mutations alter multiple cellular signaling pathways, including but not limited to PI3K, hypoxia-inducible factor (HIF), p53, MYC, and AMP-activated kinase (AMPK)-liver kinase B1 (LKB1) [19].

Even though cancer cells rely mainly on glycolysis, they are able to adapt their metabolism to changes based on the availability of nutrients in their surrounding microenvironment. Upon treatment with agents that inhibit various steps of the glycolysis pathway, cancer cells will adjust by enhancing the use of OXPHOS to meet their energy needs by increasing the influx of glutamine and glutamate in the tricarboxylic acid (TCA) cycle, and concurrently their proliferation will decrease [24]. Over the past few decades, the discovery of anticancer therapeutics has focused on several pathways, including those related to the Warburg effect [25,26,27,28,29,30]. Investigators have exploited this adaptation by identifying compounds that inhibit glycolysis in combination with agents that target OXPHOS, such as the mitochondrial complex-I inhibitors metformin, an antitype 2 diabetes drug, and phenformin. For instance, the disruption of ATP-generating pathways using complex-I inhibitors together with lactate dehydrogenase (LDH) inhibitors has been shown to be synergistically lethal in vitro and in vivo in melanoma, breast cancer, and lung cancer [31,32,33]. Other combinations tested that were able to induce synthetic apoptosis include metformin plus the hexokinase inhibitor 2-deoxyglucose (2DG) [34] or metformin plus the dehydrogenase kinase (PDK) inhibitor dichloroacetate (DCA) [35,36].

In the present in vitro study, we observed that the oligostilbene gnetin H (GH), purified in our laboratory from *Paeonia suffruticosa* seeds, displayed the characteristics of a novel glycolysis inhibitor. We also investigated its mechanism of action and the synergetic potential of using it in combination with the OXPHOS inhibitor phenformin as a possible antineoplastic therapeutic intervention.

## 2. Results

### 2.1. Extraction, Preparation, and Purification of GH from Seeds of Paeonia suffruticosa

**Figure 1:**
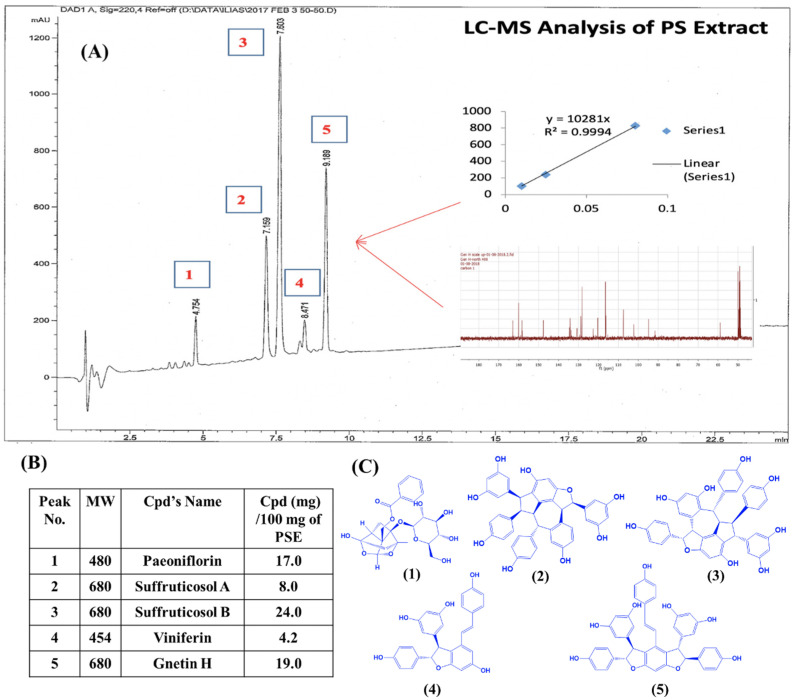
*P. suffruticosa* seed extract characterization. LC-MS analysis of the *P. suffruticosa* seed extract fractions (**A**), their relative percentage (**B**), and their chemical structures (**C**).

The peony (*Paeonia suffruticosa*) seed extract (PSE) was prepared at the National Center for National Product Research/School of Pharmacy of the University of Mississippi via 70% EtOH extraction, and fractions were detected and analyzed by LC-MS. Pure GH was prepared by LC prep/semiprep columns, and identity/purity was reconfirmed by LC/MS and NMR. Figure 1 shows the LC-MS analysis of the *P. suffruticosa* seed extract fractions (Figure 1A), their relative percentage (Figure 1B), and their chemical structures (Figure 1C). The PSE extract was found to contain 19% of GH, indicated as analyte #5 in Figure 1A,B.

### 2.2. Gnetin H Inhibits Cell Proliferation, Cell Metabolism, and Acidification of the Cell Culture Medium

**Figure 2:**
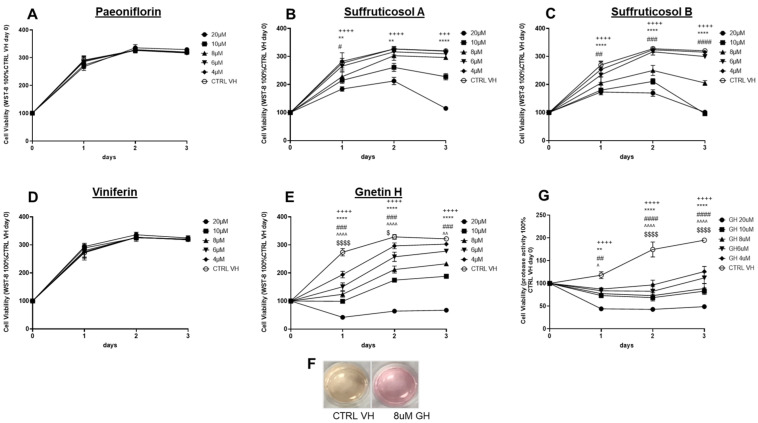
T98G cell proliferation, cell metabolism, and acidification of the cell culture medium following GH treatment. T98G cells treated with incremental concentrations (4–20 µM) of paeoniflorin (**A**), Suffructicosols A (**B**), and B (**C**), ε-viniferin (**D**), and GH (**E**) up to 3 days, and proliferation was measured by using a WST-8 cell proliferation kit). Data are representative of three biological repeats (±SEM) analyzed by 2-WAY ANOVA/Dunnett’s multiple comparisons test; +++ *p ≤* 0.001, ++++ *p ≤* 0.0001, ** *p ≤* 0.01, **** *p ≤* 0.0001, # *p ≤* 0.05, ## *p ≤* 0.01, ### *p ≤* 0.001, #### *p ≤* 0.0001, ^^ *p ≤* 0.01, ^^^^ *p ≤* 0.0001, $ *p ≤* 0.05, $$$$ *p ≤* 0.0001. (if the symbol is: +) CTRL VH vs. GH 20 µM, (if the symbol is: *) CTRL VH vs. GH 10 µM, (if the symbol is: #) CTRL VH vs. GH 8 µM, (if the symbol is: ^) CTRL VH vs. GH 6 µM, (if the symbol is: $) CTRL VH vs. GH 4 µM. GH inhibited cell culture media acidification, and the effect was more distinct after 3 days at higher concentrations (8 µM), suggesting a modulation of cell metabolism and inhibition of glycolysis (**F**). T98G cells were also tested using a cell viability assay based on protease activity, which is independent of cell metabolism (**G**). Data are representative of three biological repeats (±SEM) analyzed by 2-WAY ANOVA/Dunnett’s multiple comparisons tests; ++++ *p* ≤ 0.0001, ** *p* ≤ 0.01, **** *p* ≤ 0.0001, ## *p* ≤ 0.01, #### *p* ≤ 0.0001, ^ *p* ≤ 0.05, $$$$ *p* ≤ 0.0001. (if the symbol is: +) CTRL VH vs. GH 20 µM, (if the symbol is: *) CTRL VH vs. GH 10 µM, (if the symbol is: #) CTRL VH vs. GH 8 µM, (if the symbol is: ^) CTRL VH vs. GH 6 µM, (if the symbol is: $) CTRL VH vs. GH 4 µM.

T98G human glioblastoma cells were treated with incremental concentrations (4–20 µM) of the different PSE fractions, paeoniflorin (Figure 2A), suffructicosol A (Figure 2B), and B (Figure 2C), ε-viniferin (Figure 2D), and GH (Figure 2E), and incubated with the samples for a time period of 3 days. Proliferation of the cells was measured using the water-soluble tetrazolium salt, WST-8, which produces a water-soluble formazan dye upon reduction in the presence of an electron mediator. The amount of formazan generated by dehydrogenases is directly proportional to the number of living cells over a period of time. Suffructicosols A and B and GH induced a time-dependent reduction in cell proliferation between the concentrations of 4 and 20 µM when compared to the control samples (Figure 2B,C,E). Paeniflorin and ε-viniferin did not reduce cell proliferation at the concentrations tested (maximum of 20 µM) (Figure 2A,D).

We also observed that GH inhibited media acidification, and the effect was more distinct and visible after 3 days at higher and sublethal concentrations (e.g., 8 µM), suggesting a modulation of cell metabolism and inhibition of glycolysis (Figure 2F). Inhibition of glycolysis may result in decreased extracellular acidification, causing a reduction in cell proliferation and tumor growth [37,38,39,40]. For this reason, a protease activity assay, which is independent of cell metabolism, was also used to investigate cell viability upon treatment with GH. We observed that GH induced a time- and concentration-dependent inhibition of cell metabolism (Figure 2E, WST-8 assay) that corresponded to a delay in cell proliferation and a cytostatic effect at the highest concentrations between 4 and 20 µM (Figure 2G, protease activity assay).

### 2.3. Gnetin H Inhibits Cell Viability of Human Glioblastoma and Murine Melanoma

**Figure 3:**
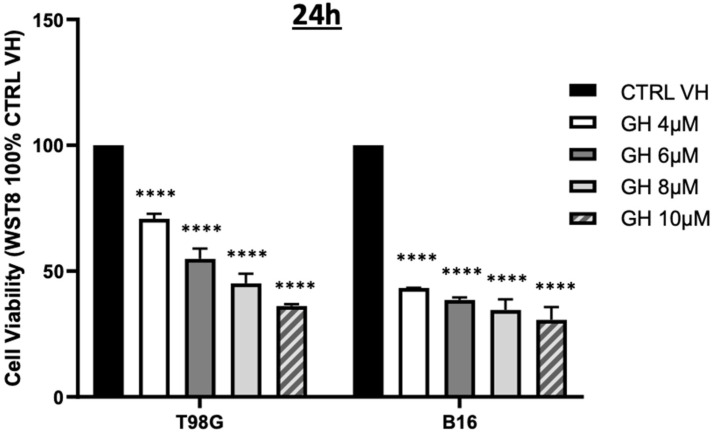
T98G and B16 cell viability following GH treatment. Human glioblastoma T98G and murine melanoma B16 cells were treated with an incremental concentration of GH (4–10 µM) for 24 h and a WST8 assay was used to determine cell viability. Data are representative of three biological repeats (±SEM) 2 WAY ANOVA/Dunnett’s multiple comparisons test; **** *p* ≤ 0.0001.

The growth-suppressive properties of gnetin H on human lung cancer, breast cancer, promyelocytic leukemia cells, and osteosarcoma have been published earlier by other researchers [10,11,12,13,14,15]. Here, we investigated the effect of GH on the proliferation of human glioblastoma T98G cells and murine melanoma B16–F10. Cells were treated with an incremental concentration of GH (4–10 µM) for 24 h, and the WST8 assay results showed that GH induced a dose-dependent inhibition of cell viability when compared to the solvent-treated control samples (Figure 3).

At the concentrations of 4 µM and 8 µM, T98G cell viability was decreased by 25% and 50%, respectively. The B16 melanoma cells were more sensitive than the T98G glioblastoma cells, and they showed a 50% decrease in viability at the lowest tested concentration of 4 µM.

### 2.4. Gnetin H Inhibits Lactic Acid Production without Affecting Glucose Transport

**Figure 4:**
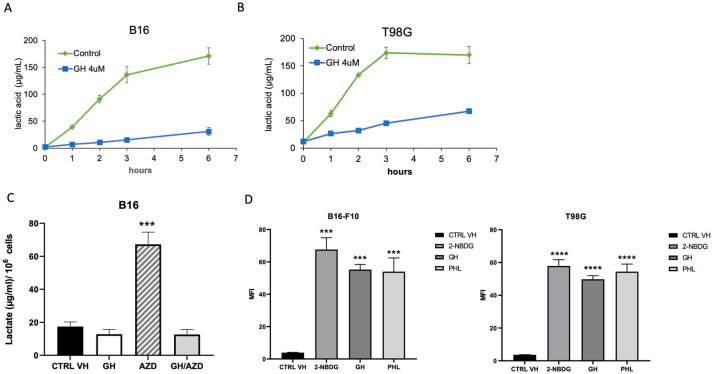
T98G and B16 intracellular and extracellular lactic acid production and glucose transport measurement. B16 (**A**) and T98G (**B**) cells were treated with 4 µM GH and the level of extracellular lactate was measured by sampling the culture medium at 0, 1, 3, and 6 h. Intracellular lactic acid levels were measured in B16 cells (**C**) following 3 h of treatment with 4 µM GH, AZD3965 (100 nM), an MCT-1 inhibitor, or cotreated with GH and AZD3965. Data are representative of three biological repeats (±SEM); one-way ANOVA Dunnet’s multiple comparisons test; CTRL VH vs. single treatment; *** *p* ≤ 0.001. Flow cytometric analysis of fluorescent glucose analog 2-NBDG (100 μg/mL) following treatment with 4 µM GH or phloretin (PHL), a glucose transporter inhibitor in B16 and T98G cells (**D**). Data are representative of three biological repeats (±SEM); (one-way ANOVA, Dunnet’s post-test, *** *p* < 0.001, **** *p* < 0.0001).

To assess if GH was able to inhibit lactate production, we treated B16 (Figure 4A), T98G (Figure 4B), and MDA-MB-231 cells (Supplementary Figure S1A) with 4 µM GH and measured the level of extracellular lactate by sampling the culture medium at 1, 2, 3, and 6 h. We detected, in all cell lines treated with GH, a significant inhibition in extracellular lactate production when compared to untreated controls, starting as early as 1 h.

Monocarboxylate transporters (MCTs) regulate the efflux of lactate from the cell along with a proton to maintain pH homeostasis [41]. To evaluate the ability of GH to inhibit MCTs and lactate excretion, we measured the intracellular lactic acid levels after GH treatment. B16 cells (Figure 4C) were treated for 3 h with 4 µM GH, AZD3965, an MCT-1 inhibitor, or cotreated with GH and AZD3965. AZD3965 treatment resulted in an almost 4-fold accumulation of lactic acid, while treatment with GH did not lead to an increase in intracellular lactic acid above control values. GH in combination with AZD3965 prevented intracellular lactic acid accumulation. Together, these results indicate that GH does not inhibit lactic acid export but rather inhibits lactic acid production in B16–F10 melanoma cells.

Blocking glucose transport is one of the possible mechanisms of glycolysis inhibition. We treated B16, T98G (Figure 4D), and MDA-MD-231 (Supplementary Figure S1B) cells with either 4 µM GH or 300 µM phloretin, a glucose transporter inhibitor, and incubated the cells in a medium containing the fluorescent glucose analog, 2-NBDG. We measured the mean fluorescence intensity of 2-NBDG by flow cytometry and observed that phloretin inhibited the accumulation of 2-NDGB by 34.78% in B16 and by 26.37% in T98G cells. Conversely, GH did not inhibit glucose uptake across the cell membrane in the cell lines tested.

### 2.5. Gnetin H Exhibits Significant Cytotoxicity when Combined with a Mitochondrial Complex-I Inhibitor and Induces Apoptosis

**Figure 5:**
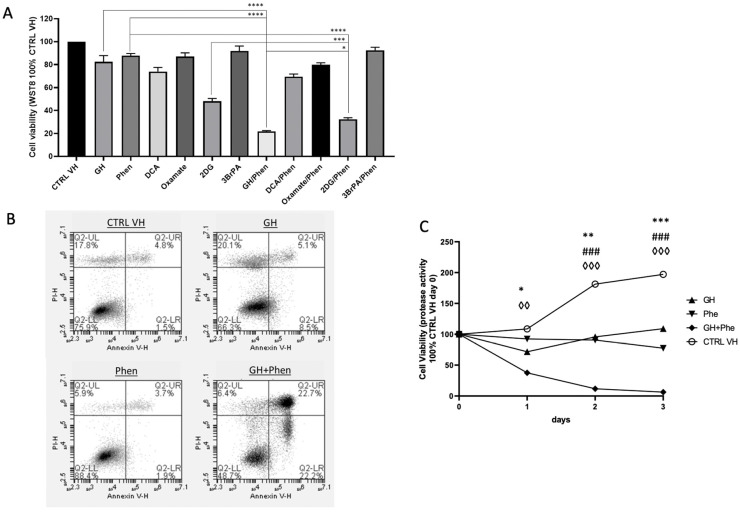
Effects on T98G cells of combined treatment with glycolysis inhibitors and the mitochondrial complex-I inhibitor phenformin. (**A**) T98G cells treated for 48 h with either 4 µM GH or other known glycolysis inhibitors, such as 25 mM dichloroacetate (DCA), 25 mM oxamate, 25 mM 2-deoxy-d-glucose (2DG), 25 µM 3- bromopyruvate (3BrPA), alone or in combination with 100 µM of the mitochondrial complex-I inhibitor phenformin. Data is representative of three biological repeats (±SEM) 2-way ANOVA/Tukey’s multiple comparisons test; * *p* ≤ 0.05, *** *p* ≤ 0.001, **** *p* ≤ 0.0001 (**B**) Assessment of apoptosis (early/late apoptosis) and necrosis following treatment with 4 µM GH alone or in combination with 100 µM phenformin for 48 h by annexin-V/propidium iodide (PI) staining. (**C**) Analysis of T98G viability independent from the effect on cell metabolism using a protease activity-based cell viability assay. T98G cells were treated for 3 days with 8 µM GH, 100 µM phenformin, or the combination of the two was assessed at 24 h. Data is representative of three biological repeats, analyzed by 2-WAY ANOVA/Dunnett’s multiple comparisons test; CTRL VH vs. GH/time point, * *p* ≤ 0.05,** *p* ≤ 0.01, *** *p* ≤ 0.001; CTRL VH vs. Phen/time point, ### *p* ≤ 0.001; CTRL VH vs. GH + Phen/time point, ◊◊ *p* ≤ 0.01, ◊◊◊ *p* ≤ 0.001.

The results presented thus far suggest that gnetin H inhibits glycolysis. Suppression of glycolysis together with oxidative phosphorylation (OXPHOS) represents a potentially efficient strategy for targeting cancer metabolism [31,32,33,34,35,36]. In order to investigate if GH could assist inhibitors of OXPHOS to induce cell death, we treated T98G (Figure 5A), B16 (Supplementary Figure S2A), and MDA-MB-231 (Supplementary Figure S2B) for 48 h with either 4 µM GH or other known glycolysis inhibitors, such as 25 mM dichloroacetate (DCA), 25 mM oxamate, 25 mM 2-deoxy-d-glucose (2DG), 25 µM 3-bromopyruvate (3BrPA) [36,42,43,44], alone or in combination with the mitochondrial complex-I inhibitor 100 µM phenformin [45]. The concentrations of known glycolysis inhibitors were determined from previously published literature sources. Additionally, 2DG (25 mM) decreased the viability of the cells slightly more than GH (4 µM). However, GH (4 µM) had a more cytotoxic effect than the other glycolysis inhibitors when they were used in combination with phenformin (100 µM).

To assess if programmed cell death was involved in the reduction of cell viability measured by WST-8, we treated T98G with GH alone (4 µM) or in combination with Phen (100 µM) for 48 h and stained the cells with annexin-V/propidium iodide (PI). We observed that the combination treatment with GH and an OXPHOS inhibitor (Phen) induced early and late apoptosis when compared to GH or Phen alone, as these treatments caused a nonsignificant increase in early apoptosis (Figure 5B).

To evaluate the effect of GH, phenformin, and their combination on cell proliferation independently from the effect of cell metabolism, we performed a protease activity-based cell viability assay (Figure 5C). We treated T98G for 3 days with either GH alone (8 µM) or Phen alone (100 µM), or the combination of these two compounds. We observed that GH and Phen, when used individually, displayed merely a cytostatic effect, while their combination induced a severe reduction of cell viability starting at 24 h, consistent with the induction of apoptosis, as seen in Figure 5B.

### 2.6. Gnetin H Is a Potent Inhibitor of Glycolysis as Measured by Extracellular Acidification Rate Analysis

**Figure 6:**
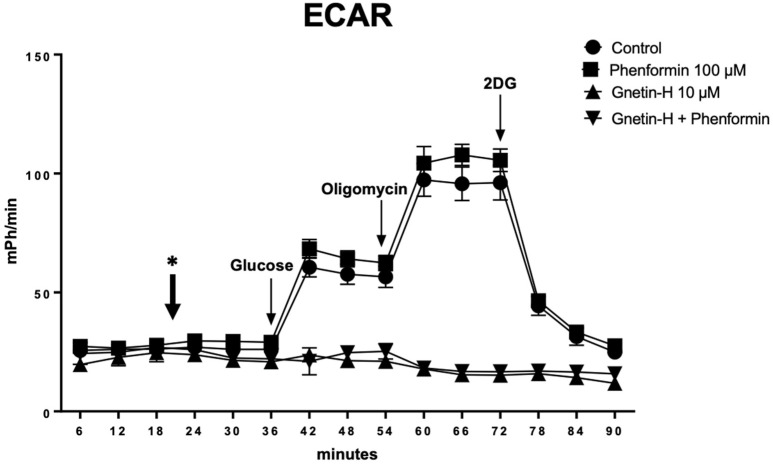
The treatment with GH impairs the glycolytic activity of T98G cells. The glycolytic activity was measured by Agilent SeaHorse XFe96 Analyzer after 20 min of treatment with either phenformin (100 µM), gnetin H (10 µM), or the combination injected into the cell media following basal ECAR measurements. (*) indicates the time of treatment with phen, GH, or the combination. ECAR data are shown as mpH/min normalized to proteins and represent the mean ± SD of *n* = 5 independent measurements under normal cell culture conditions. Glycolytic profiles were obtained using the Agilent SeaHorse Glycolysis Stress Test. The ECAR was measured under glucose starvation and after the sequential addition of glucose (basal glycolysis), oligomycin (maximal glycolysis or glycolytic reserve), and 2-DG (glycolysis specificity).

The impact of GH treatment on cell glycolytic activity was more deeply investigated by performing a glycolysis stress test using the Seahorse XFe96 Flux Analyzer. As shown in Figure 6, GH alone was effective in preventing the increase in ECAR (i.e., glycolytic rate) induced by glucose addition in T98G cells, and the maximal increase in ECAR induced by oligomycin (i.e., glycolytic reserve).

### 2.7. RNA-Seq Shows That Gnetin H and the OXPHOS Inhibitor Phenformin Induce a Decrease in TXNIP Gene Expression

To study the effect of gnetin H on gene expression, we conducted RNA-Seq analysis on T98G cells treated with GH, phenformin, and 2DG alone, compared to the combination of GH with phenformin or 2DG with phenformin. T98G cells were treated with 8 µM GH, 25 mM 2DG, or 100 µM phenformin as single agents, or combinations of GH plus phenformin, or 2DG plus phenformin, for the duration of 2 h. Cells were also exposed to DMSO (solvent control) for the same duration. RNA-Seq libraries were prepared in triplicate for each treatment and were sequenced. The sequencing run produced high-quality reads, with a median quality score of Q > 30 and an average of 20 million reads per sample. Genes exhibiting significant differential expression (*p*-value of ≤0.05 and fold-change of ≥2) between compound-treated and solvent-treated cells were identified (Supplemental Table S1).

**Figure 7:**
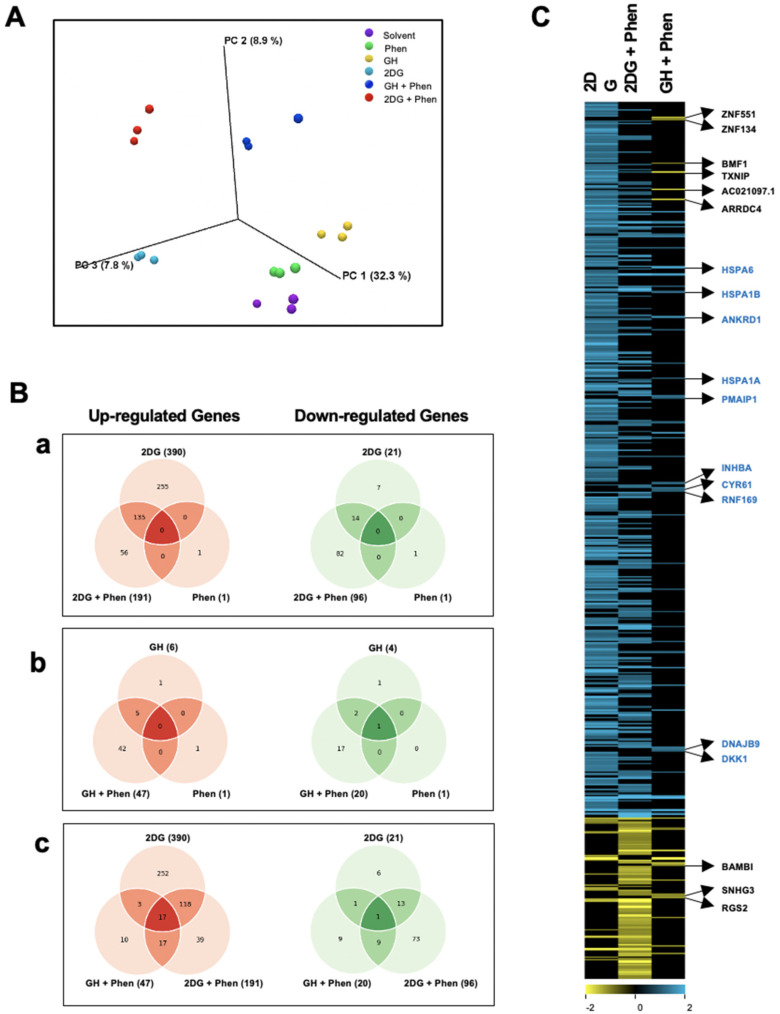
RNA-Seq analysis of T98G cells treated with 2DG, Phen, 2DG + Phen, GH, or GH + Phen. (**A**) The experimental variation between RNA-Seq experiments performed on 3 biological replicate samples was analyzed with a PCA plot. (**B**) Venn diagrams comparing the number of up- or downregulated genes that significantly responded (≥2-fold induction, *p*-value *≤* 0.05) to the different treatments. (**C**) Hierarchical cluster analysis was performed on 566 genes that displayed a ≥2-fold induction (*p*-value *≤* 0.05) response to at least one of the three treatments compared to the solvent control. Blue and yellow colors represent up- and downregulated genes, respectively. The genes listed responded to GH + Phen treatment, but not to 2DG or 2DG + Phen treatment.

Principal component analysis (PCA) revealed that the samples within each treatment group clustered together and that there was good reproducibility among the three replicate samples in each group (Figure 7A). The samples treated with GH alone or with phenformin alone clustered near those treated with solvent, indicating that these two individual treatments did not have a strong impact on the cells. This is most likely due to the low concentrations used for each of these two treatments, and the short duration of the experiment.

This result was also evident in the distribution of the number of genes that responded to each treatment, as shown in the Venn diagrams (Figure 7B). While phenformin treatment caused the upregulation of only one gene and the downregulation of another gene (Figure 7B, panel a).

Interestingly, GH treatment caused the upregulation of six genes and the downregulation of four genes (Figure 7B, panel b). However, PCA analysis revealed that the 2DG treatment and the two combination treatments generated responses that were different from those of the solvent and also different from each other (Figure 7A). A substantial number of genes responded to treatment with 2DG, with 390 genes upregulated and 21 downregulated. Additionally, the combination treatment of T98G cells with 2DG plus phenformin yielded a substantial upregulation of 191 genes and a downregulation of 96 genes, while the treatment with GH plus phenformin resulted in the upregulation of 47 genes and the downregulation of 20 genes. In addition, the combination treatments showed several genes that were not present in the respective single treatments of the two compounds used (Figure 7B, panels a, b), indicating that there were several unique responses to the combination treatments.

Since the largest number of regulated genes was observed with the 2DG treatment and the two combination treatments, we further explored and compared the distribution of genes across these three treatments. This analysis revealed that only 17 upregulated genes and 1 downregulated gene were commonly shared between the three treatments (Figure 7B, panel c).

To further investigate the transcriptional responses observed following 2DG, 2DG plus phenformin, and GH plus phenformin treatments, we performed hierarchical cluster analysis on a set of 566 genes that were affected in response to at least one of these three treatments (Figure 7C). Responses to the three treatments were quite different from each other. While there were several overlapping genes in the analysis of 2DG and 2DG plus phenformin treatments, we observed that many of the genes induced by 2DG were not induced by the 2DG plus phenformin treatment. In addition, there were several genes that were downregulated by 2DG plus phenformin, but not by 2DG alone (Figure 7C). Gene regulation observed after GH plus phenformin treatment was less significant. However, it showed several genes that did not respond to 2DG or to 2DG plus phenformin. Interestingly, many of the regulated genes play functional roles in protein folding, including HSPA6, HSPA1A, HSPA1B, and DNAJB9 (Figure 7C).

To gain insight into the functional roles of the responding genes, we further analyzed the transcriptional responses to 2DG, 2DG plus phenformin, and GH plus phenformin by gene ontology (GO) enrichment analysis. Supplemental Table S2 shows the top 15 significantly enriched functional categories from each analysis conducted using gene lists with a substantial number of genes (for gene lists with <25 genes, no significantly enriched functional categories were identified). As would be expected, the over-represented functional categories (*p*-value *≤* 0.05) for 2DG-induced genes included “regulation of glycolytic process”, “regulation of ATP metabolic process” and “response to glucose starvation”. For the 2DG plus phenformin upregulated genes, we found some functional categories that overlapped with those seen in the 2DG treated cells (e.g., protein kinase B signaling, and ER stress), and in addition, there were several functional categories that did not overlap with 2DG (e.g., polyamine catabolism, ERK1 and ERK2 cascade, GPCR signaling, and steroid metabolism). For the 2DG plus phenformin downregulated genes, the most enriched functional categories were related to transcriptional regulation and RNA metabolism. Interestingly, for the GH plus phenformin upregulated genes, the enriched functional categories included “heat acclimation”, “protein refolding”, and “regulation of unfolded protein response”. These results further demonstrate that the transcriptional responses to 2DG and to the two combination treatments are quite distinct from each other.

Previous transcriptomic studies have investigated the effects of metformin [46], which allowed us to investigate and determine whether genes that are known to frequently respond to metformin also responded to the treatments used in the present study. We observed that, among the 21 genes known to be frequently upregulated by metformin [46], 7 genes responded to at least one of the treatments in our study. Interestingly, these genes were not upregulated by phenformin alone, most likely due to the low dose (100 µM) and short duration of exposure (2 h) used in our study, but their expression was induced when phenformin was combined, under the same experimental conditions, with either 2DG or GH (Supplemental Table S3) [46]. These results demonstrate that both 2DG and GH potentiate the transcriptional responses observed following phenformin treatment in a rapid manner. Among the downregulated genes, the two genes TXNIP and ARRDC4, which are known to play functional roles in glucose uptake, are frequently found to be downregulated by metformin [46]. In our study, AARDC4 was not affected by phenformin alone but was approximately 5-fold downregulated by GH plus phenformin. Regarding TXNIP, we did observe the downregulation of TXNIP by phenformin alone, and this downregulation was strongly potentiated by the combo treatment of GH plus phenformin. Interestingly, both TXNIP and ARRDC4 were strongly downregulated by GH alone, further confirming our results from biochemical analyses that GH may play a role in regulating glucose metabolism.

### 2.8. Gnetin H Substantially Reduces TXNIP Protein Expression and Both GH and 2DG in Combination with Phenformin Enhances p-AMPK

**Figure 8:**
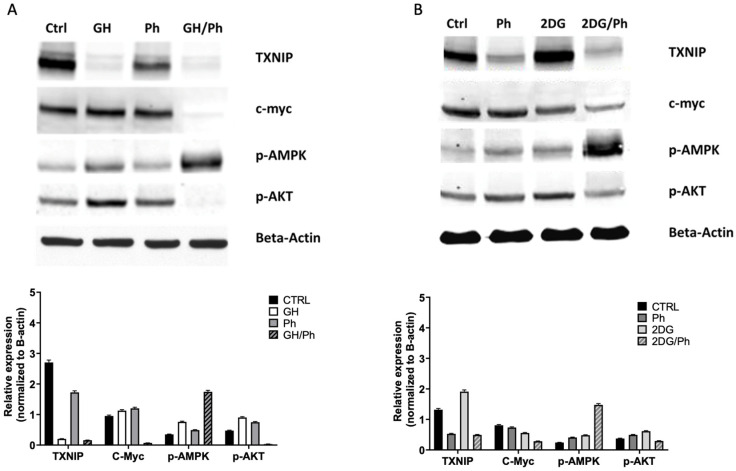

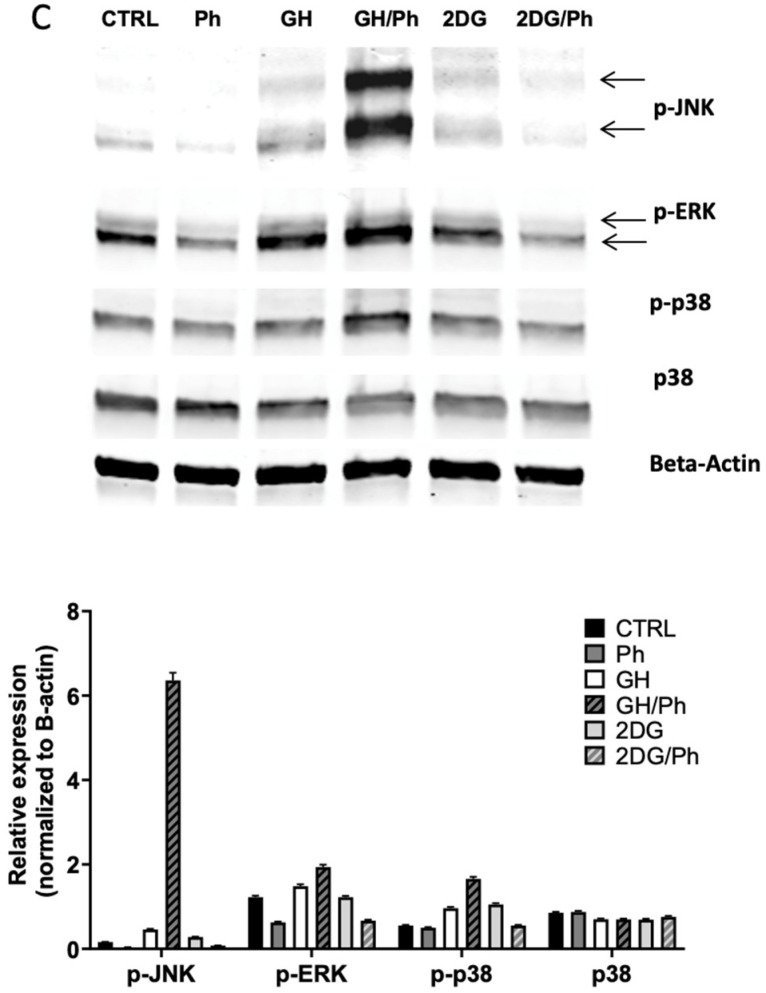
Western blot expression analysis of key mediators of pathways involved in the regulation of cell metabolism following GH treatment in T98G cells. (**A**) Treatment of samples (alone or in combination with GH and phenformin) is indicated above the protein bands. (**B**) Treatment of samples (alone or in combination with phenformin and 2DG) is indicated above the protein bands. Antibodies (TXNIP, c-myc, p-AMPK, and p-AKT) are indicated on the side, *β*-Actin was used as the internal standard. Data is representative of three biological repeats. (**C**) Treatment of samples (alone or in combination with phenformin, GH, and 2DG) is indicated above the protein bands. Antibodies (p-JNK, p-ERK, p-p38, p38) are indicated on the side, *β*-Actin was used as the internal standard. Data are representative of three biological repeats.

A western blot analysis was performed on T98G cells to confirm the RNA-Seq data (Figure 8A–C). As expected, we observed a marked decrease in TXNIP protein expression following 3 h of treatment with GH alone, while 2DG slightly increased expression (Table 1 and Table 2). Phenformin treatment alone also slightly reduced TXNIP protein levels.

Additionally, we measured the protein levels of key mediators of pathways involved in the regulation of cell metabolism. We observed that treatment with GH or 2DG alone slightly increased p-AMPK levels, while GH or 2DG in combination with phenformin substantially enhanced p-AMPK protein levels. GH in combination with phenformin substantially reduced both c-myc and p-AKT protein levels. Levels of c-myc were also reduced by 2DG in combination with phenformin.

GH plus phenformin substantially increased the levels of p-JNK, while 2DG plus phenformin did not. GH alone enhanced p-ERK levels, and when combined with phenformin, p-ERK levels were further enhanced. In contrast, p-ERK levels were reduced by 2DG plus phenformin. GH plus phenformin enhanced p-p38, while 2DG plus phenformin did not (Figure 8C and Table 3).

## 3. Discussion

Cancer cells are characterized by uncontrolled growth, survival advantages, and metastatic potential. Neoplastic cells display an altered metabolism that provides them with a selective advantage by supporting uninterrupted growth. High-rate glycolytic flux supports cancer cells ability to generate building blocks and ATP for rapid tumor growth [17,19,22,47,48].

Gnetin H extracted from *P. suffruticosa* seeds can suppress the growth of various cancer cells in vitro [10,11,12,13,14,15]. We isolated and identified five compounds from a peony seed ethanolic extract: paeoniflorin, a monoterpenoid glycoside, and the oligostilbenes ε-viniferin, suffructisols A and B, and gnetin H.

Using the human glioblastoma cell line T98G, we tested the effect of these compounds on cell proliferation using escalating doses in time-course experiments. We observed that the resveratrol trimers suffructisols A and B, and gnetin H, were able to induce a dose- and time-dependent reduction in T98G proliferation as measured by a tetrazolium salt-based proliferation assay. Additionally, we observed that gnetin H was the only compound that inhibited media acidification at sublethal doses, showing the signature of a glycolysis inhibitor. This observation led us to investigate the anticancer activity of gnetin H using an assay that measures the activity of intracellular proteases, which is independent of cellular metabolism. By protease assay, we observed that GH dramatically decreased cell growth and that it had a cytostatic effect at the concentrations of 8 and 10 µM.

By treating additional cell lines, such as the murine B16 melanoma cells, we observed that the level of sensitivity, rather than the effect of GH is cell-line dependent. The highly glycolytic melanoma cell line B16 had an IC_50_ at 24 h of 4 µM, while the glioblastoma T98G was more resistant to an IC_50_ of 8 µM.

Cancer cells display a glycolytic phenotype with increased utilization of glucose and concomitant synthesis of lactate. Glycolytic flux results in increased lactate levels, which lead to the acidification of the cell culture medium [49]. We first studied in a time-course experiment the effect of the lowest dose of GH on B16 and T98G cells, and we detected, starting as early as 1 h post-treatment, a strong reduction in the extracellular lactic acid when compared to the solvent control. To maintain a sustained rate of glycolysis, lactate generated in glycolytic flux is exported by monocarboxylic transporters (MCT). Selective inhibition of MCTs has the potential to inhibit glycolysis in many cancer types [50], therefore we hypothesized that GH could inhibit the transport of lactate in the culture medium, causing an accumulation of intracellular lactic acid. Our data clearly demonstrated that GH does not inhibit lactate export in a cotreatment with ADZ3965, an MCT-1 inhibitor, but rather inhibits lactic acid production in B16 cells, whereas GH treatment did not. Since GH prevented the accumulation of lactic acid when combined with ADZ3965, it suggests that GH inhibits lactic acid production.

Transport of glucose uptake is the first step occurring in glycolysis [51]. It has been reported that targeting the uptake of glucose may efficiently inhibit glycolysis [19]. We demonstrated that GH does not inhibit the uptake of glucose in B16, and T98G, as shown by flow cytometry using the glucose analog 2-NBDG. Phloretin, an inhibitor of glucose transporters, effectively reduced the uptake of glucose when compared to the control samples, and treatment with GH did not show any effect on glucose transport. Collectively, these findings suggest that GH inhibits glycolysis by targeting an enzymatic reaction within the glycolysis pathway and that this inhibits lactic acid production.

Several reports suggest that targeting glycolysis may be used as a potential therapeutic approach either alone or in combination with existing therapies for cancer treatment [31,33,39,52,53,54,55]. Induction of metabolic catastrophe in cancer cells through the targeting of metabolic pathways is conceived as an attractive strategy to uproot tumors, which are resistant to chemotherapeutic regimens. Additionally, it has been proposed that targeting cellular metabolism may improve the response to cancer therapeutics and may devise strategies to overcome drug resistance in cancer therapy [56,57,58].

Recent studies focused on energy metabolism rewiring in cancer cells have shown promising results both in vitro and in vivo [31,33,55,59]. The biguanides metformin and phenformin inhibited mitochondrial complex-1 and showed antitumor effects [55,60,61,62]. Simultaneous targeting of glycolysis and mitochondrial complex 1 leads to cell death by targeting the metabolic plasticity of cancer cells. It has been demonstrated that inhibition of glycolysis pushes cancer cells to switch from glycolysis to OXPHOS for ATP generation depending on the availability of oxygen and nutrients [63]. This prompted us to investigate metabolic homeostasis by treating cancer cells with GH and other metabolic inhibitors in combination with an inhibitor of OXPHOS. Targeting metabolic pathways with glycolytic inhibitors, such as 2-DG, 3-bromopyruvate, DCA, oxamate, and drugs interfering with the reducing equivalents of cells have shown promising outcomes in preclinical studies [59,64]. In addition, targeting the lactate generation pathway in combination with mitochondrial inhibitors is appealing, especially in highly glycolytic cancers.

We demonstrated that treatment with 2DG alone mostly affected cancer cell viability when compared to GH and the other glycolytic inhibitors. Interestingly, the combination treatment of GH and phenformin was highly cytotoxic when compared to 2DG/phenformin. Miskimins et al. suggested that the simultaneous inhibition of glycolysis and complex I induced apoptosis in cancer cells. However, apoptosis mostly depends on the p53 status of the cells [64]. We showed that treatment with phenformin or GH alone had a negligible effect on early apoptosis, while the combination of the two active compounds strongly induced early and late apoptosis. By protease assay, we also observed that treatment with either GH or phenformin alone had a cytostatic effect, while their combination exhibited a strong synergistic effect and led to a statistically significant decrease in cell viability.

Although specific energy and nutrient sensors, such as AMP-activated protein kinase (AMPK), act as the protective response to nutrient deprivation conditions, AMPK activation might target signaling pathways associated with tumorigenesis and inhibit tumor progression. We report that AMPK, upon combination treatment of GH and phenformin, initiates a signaling cascade resulting in the activation of pAMPK, pJNK, and pERK and p-p38 pathways with concomitant induction of cell death in glioblastoma cells. In addition, enhanced p-AMPK levels are considered a marker of metabolic catastrophe and ATP depletion [65,66].

To the best of our knowledge, we are the first research group to present evidence supporting the role of gnetin H as a glycolysis inhibitor. We demonstrated that the treatment of cancer cells with GH purified from peony seeds is not only cytostatic but also simultaneously reduces the synthesis of lactic acid and the expression of TXNIP. Our data suggest that GH, when used in combination with an OXPHOS inhibitor, such as phenformin, could treat various cancers through metabolic modulation, as this combination induces metabolic catastrophe and apoptosis. Our current study involving this novel combination treatment could pave the way for future preclinical and clinical studies to investigate the potential of these compounds for therapy-resistant cancers.

## 4. Materials and Methods

### 4.1. Cell Lines and Culture Conditions

Human glioblastoma (T98G), and murine melanoma cell lines (B16–F10) were obtained from the American Type Culture Collection (Manassas, VA, USA). Cells were routinely cultured in Dulbecco’s Modified Eagles Medium (DMEM) supplemented with 10% heat-inactivated fetal bovine serum (Hyclone, Logan, UT, USA), penicillin (100 U/mL), streptomycin (100 μg/mL) (Invitrogen Life Technologies, Waltham, MA, USA), and maintained at 37 °C in a 5% CO_2_ humidified incubator (Thermo-Fisher Scientific, Waltham, MA, USA).

### 4.2. Gnetin H Extraction and Purification and Other Reagents

Dichloroacetate (DCA), oxamate, 2-deoxy-d-glucose (2DG), 3-bromopyruvate (3BrPA), and phenformin (Phen) were purchased from Cayman Chemicals (Ann Arbor, MI, USA). The oligostilbene trans-gnetin H (GH) was purified from *P. suffruticosa* seeds, authenticated at the NCNPR and School of Pharmacy, UM. *P. suffruticosa* extract was prepared via 70% EtOH extraction. Pure GH was prepared by LC prep/semiprep columns, and identity/purity reconfirmed by LC/MS and NMR. Quantitative LC analysis was conducted using an Agilent 1100 LC system controlled by Chemstation software v. L01.11 (Agilent Technologies, Santa Clara, CA, USA). The analysis was carried out on RP-C18 column with column oven temperature set at 25 °C and using the gradient system of HPLC grade eluent water (A) and acetonitrile (B) at a flow rate of 1.0 mL/min and injection volumes of 50–100 µL at wavelengths of 254, 280, and 325 nm. Acetic acid was added as a modifier to achieve a final concentration of 0.1% in each solvent. A stock of standard solution of high-purity marker compound was prepared in methanol as an appropriate solvent to ensure complete solubility. Serial dilutions were prepared from the stock solutions to create a calibration curve. Linear regression equation (y = mx + c) was calculated by the least-square regression method to determine the linearity.

### 4.3. Cell Viability: WST-8 Assay

T98G cells (3000 cells/well), and B16-F10 cells (1000 cells/well) were seeded in 384-well tissue culture-treated microplates (Falcon, Franklin Lakes, NJ, USA). After 24 h, cells were treated with either solvent (PBS or DMSO) control, GH (4–10 µM), Phen (100 µM), DCA, oxamate, 2DG (25 mM), 3BrPA (25 µM) or the combination of drugs and Phen using an Agilent Bravo automated liquid handling system (Agilent Technologies, Santa Clara, CA, USA). Cells were treated either for 24, 48 h or over a time course from day 0 up to day 3. WST-8 reagent (Sigma–Aldrich, St. Louis, MO, USA) was added to each well as per manufacturer’s instructions and incubated for 2 h at 37 °C. The absorbance was measured at 450 nm using a Spectramax M2e microplate reader (Molecular Devices, San Jose, CA, USA).

### 4.4. Cell Viability: Protease Assay

T98G cells (3000 cells/well) were seeded in 384-well tissue culture treated black-walled, clear-bottom microplates (Corning Life Sciences, Tewksbury, MA, USA). After 24 h, cells were treated with either solvents (PBS or DMSO) control, GH, Phen, or the combination of GH and Phen using an Agilent Bravo automated liquid handling system (Agilent Technologies, Santa Clara, CA, USA). CellTiter-Fluor™ Cell Viability Assay (Promega, Madison, WI, USA, Cat.# G6080) was performed following manufacturer protocol to measure live-cell protease activity in cells treated from day 0 to day 3. Briefly, cells were treated with drugs in 25 µL and, at indicated time points, an equal volume of GF-AFC substrate was added. The cleaved compound generates a fluorescence signal proportional to the number of living cells. After 30 min of incubation at 37 °C, the signal was measured using a Spectramax M2e microplate reader (380–400 nm Ex/505 nm Em) (Molecular Devices, San Jose, CA, USA).

### 4.5. Extracellular Lactic Acid Measurement

Lactate measurement in cell extracts or in culture medium was estimated, as described previously [41]. In brief, B16–F10 and T98G cells were plated in tissue culture-treated 96-well plates (Corning Life Sciences, Tewksbury, MA, USA) at a density of 1.5 × 10^4^ cells/well. After 24 h, the media was replaced with fresh growth media containing the solvent control (DMSO) or GH (4 µM). Media was collected at incremental time points, and lactate concentration was determined by an enzymatic assay, which results in a colorimetric (570 nm) product, proportional to the lactate present in the medium. The assay was performed using a commercially available kit (MAK064, Sigma-Aldrich, St. Louis, MO, USA), according to the manufacturer’s instructions. Experiments were performed in triplicate for each treatment (*n* = 3).

### 4.6. Intracellular Lactic Acid Measurement

For the intracellular lactate measurement, the B16-F10 cells (1.5 × 10^4^ cells/well) were plated in tissue culture-treated 96-well plates (Corning Life Sciences, Tewksbury, MA, USA). After 24 h the media was replaced with fresh media containing the vehicle control (DMSO), GH (4 µM), AZD (AZD3965, MCT-1 inhibitor), or GH/AZD for 3 h. Cells were washed 3× with PBS and lysed in 100 mM Tris-EDTA (TE) buffer containing 0.01% NP-40 followed by three freeze-thaw cycles. Samples were filtered with a 10 KDa filter, and the lactate concentration in the cell lysates was determined by an enzymatic assay, which results in a colorimetric (570 nm) product, proportional to the lactate present. The assay was performed using a commercially available kit (cat#MAK064, Sigma-Aldrich, St. Louis, MO, USA), according to the manufacturer’s instructions. Values were normalized to protein contents present in the samples. Experiments were performed in triplicate for each treatment (*n* = 3).

### 4.7. Seahorse Extracellular Flux Analysis

Glycolytic capacity was assessed by extracellular flux analysis using the Seahorse XFe96 Analyzer (Agilent, Santa Clara, LA, USA). T98G cells were seeded into a 96-well Seahorse culture plate overnight (10,000 cells per well) in DMEM culture medium. Cells were nutrient-starved in basal Seahorse medium for 1 h at 37 °C in a non-CO_2_ incubator prior to assays. Glycolysis was assessed by changes in the extracellular acidification rate (ECAR) using the glycolysis stress test kit (Agilent#103020-100). Either phenformin (100 µM), gnetin H (10 µM), or the combination was injected into the cell medium following basal ECAR measurements. Glucose was then injected into the medium (10 mM) to assess basal glycolysis, followed by oligomycin (1 µM) to measure maximal glycolysis, and finally 2-deoxyglucose (50 mM) to measure glycolysis specificity.

### 4.8. 2-NBDG Uptake Assay

T98G cells (1.0 × 10^5^ cells/well) were plated in a 24-wells plate (Corning flat bottom tissue culture treated plates, Corning Life Sciences, Tewksbury, MA, USA). After 24 h, the cell culture medium was removed, and cells were treated with 100 µg/mL of 2-NBDG (2-(N-(7-nitrobenz-2-oxa-1,3-diazol-4-yl) amino)-2-deoxyglucose, Thermo-Fisher Scientific) alone or in combination with GH, or phloretin, in 400 µL of DMEM glucose free, supplemented with sodium pyruvate, glutamax, p/s, and 0.5% FBS. Cells were incubated at 37 °C with 5% CO_2_ for 1 h. Subsequently, cells were trypsinized and washed with 1× FACS buffer. Cells were acquired with a BD FACSCalibur flow cytometer (Becton Dickinson, Mountain View, CA, USA). Forward Scatter (FSC) vs. Side Scatter (SSC) plots were used to exclude dead cells and nonspecific cellular debris. The mean fluorescence intensity (MFI) of 2-NBDG was measured, and the data were analyzed using the CellQuest Pro software v. 5.1 (BD Biosciences, Franklin Lakes, NJ, USA).

### 4.9. Detection of Apoptosis by Annexin-V/Propidium Iodide Staining

To evaluate apoptotic cell death, we treated T98G cells with GH 4 µM, phenformin 100 µM (Cayman Chemicals, Ann Arbor, MI, USA) alone or in combination, for 48 h. Cells were stained with Annexin V-FITC and propidium iodide (PI) according to the manufacturer’s protocol (Clontech, Palo Alto, CA, USA) and analyzed by flow cytometry. Samples were acquired with BD FACSCalibur (Becton Dickinson, Mountain View, CA, USA), and the data was analyzed using the CellQuest Pro software (BD Biosciences, Franklin Lakes, NJ, USA).

### 4.10. RNA-Seq Analysis

T98G cells were treated with solvent (DMSO), 2DG (10 mM), Phen (100 μM), GH (8 μM), 2DG (10 mM) + Phen (100 μM), or GH (8 µM) + Phen (100 μM) for 2 h. After treatment, cells were washed twice with PBS buffer and resuspended in the lysis buffer provided in the Qiagen RNeasy Mini Kit (Qiagen Inc., Valencia, CA, USA). Cells were homogenized for 30 s at 25,000 rpm using a PT3100 polytron (Brinkmann Instruments Inc., Westbury, NY, USA). Subsequent RNA isolation steps were performed according to the kit instructions, and an on-column RNase-free DNase I treatment was included during the procedure. The RNA concentration was determined spectrophotometrically, and the RNA quality was assessed on the Agilent 2100 Bioanalyzer (Agilent Technologies, Santa Clara, CA, USA). Sequencing libraries were generated with 3 µg of total RNA using the Illumina TruSeq Stranded mRNA Sample Preparation Kit. The libraries were assessed for size and purity using the Agilent 2100 Bioanalyzer and quantitated by qPCR using a KAPA Biosystems Library Quantitation kit. Libraries were normalized, pooled, and diluted to a loading concentration of 1.8 pM, loaded on an Illumina High-Output Flow Cell, and sequenced on the Illumina NextSeq 500 instrument (Illumina, San Diego, CA, USA). The sequencing run generated ~20 million 150-bp paired-end reads per sample, and all run metrics, including cluster densities and total sequence yields, were within the recommended parameters.

Data analysis was performed using the Qiagen CLC Genomics Workbench (CLCGxWB) version 11 software. Quality assessment of the reads indicated that the average median score at each base across the reads in each sample was Q > 30. The reads were mapped to the *H. sapiens* reference genome (GRCh38 assembly, Ensemble Release 89), using default parameters with a strand-specific alignment protocol. The mapping report indicated that all metrics fell within the recommended parameters, with >80% of the reads per sample mapping to the reference genome. The mapping results generated values for fragments per kilobase of exon per million reads mapped (FPKM) for each gene. The FPKM values were used to generate a PCA plot with CLCGxWB software, to assess the relatedness among the samples. The “Differential Expression for RNA-Seq” tool in CLCGxWB software was used, using default parameters, to identify differentially expressed genes between compound-treated and solvent-treated samples. Genes that had a fold-change of ≥2, a Bonferroni-corrected *p*-value of ≤0.05, and a maximum group mean FPKM of ≥1 (i.e., the maximum of the average FPKM across the two groups in each statistical comparison) were considered to be significantly differentially expressed. Gene ontology (GO) enrichment analysis was conducted to distribute the responding genes into GO-based biological process categories using the PANTHER classification system [67] available at the Gene Ontology Consortium website (geneontology.org). Hierarchical cluster analysis was performed using Gene Cluster 3.0 [68] with the Pearson correlation similarity metric and the average linkage clustering method, and the resulting heat map was generated using Java Tree View [69].

RNA-Seq data accession number: The RNA-Seq data described in this article are accessible through accession No. GSE224692 at NCBI’s Gene Expression Omnibus database.

### 4.11. Immunoblotting

Whole-cell lysates were prepared, and immunoblotting was performed as described previously [41]. In brief, the cells were washed with ice-cold PBS x1 and lysed in ice-cold RIPA lysis buffer supplemented with protease and phosphatase inhibitors. The cell lysates were centrifuged at 13,000× g for 20 min and supernatants were used for immunoblotting. Equal amounts of protein samples (50 μg) were resolved on 4–15% SDS-polyacrylamide gel and then transferred onto a nitrocellulose membrane (BIO-RAD, Hercules, CA, USA). The membranes were blocked with TBS blocking buffer (LI-COR, Lincoln, NE, USA) and probed with antibodies against pJNK, pERK, pAMPK, p-P38, p38, c-myc and TXNIP diluted 1:1000 (Cell Signaling Technology, Inc., Danvers, MA, USA). Anti-β-actin antibody was used as housekeeping control on membranes that were stripped of the other primary antibodies (Cell Signaling Technology Inc., Beverly, MA, USA). After washing, the membranes were incubated with HRP-conjugated secondary antibodies diluted 1:4000 (Santa Cruz Biotechnology, Paso Robles, CA, USA) and detected by chemiluminescence using a Li-Cor Odyssey scanner (LI-COR Biosciences, Lincoln, NE, USA).

### 4.12. Statistical Analysis

Values are represented as mean ± standard error (S.E.). A TWO-WAY ANOVA/Tukey’s multiple comparisons test or a two-way ANOVA/Dunnett’s multiple comparisons test were performed using a commercially available software GraphPad Prism 9.0 (GraphPad Software Inc, San Diego, CA, USA) and the *p*-value ≤ 0.05 was considered significant.

## 5. Conclusions

We presented evidence supporting the role of gnetin H as a glycolysis inhibitor and demonstrated that treatment of cancer cells with GH purified from peony seeds reduces the synthesis of lactic acid and the expression of TXNIP and is cytostatic. Combined treatments of GH with an OXPHOS inhibitor, such as phenformin, induce metabolic catastrophe and apoptosis, paving the way for future preclinical and clinical studies to investigate the potential of these compounds for therapy-resistant cancers.

## Figures and Tables

**Table 1:** Densitometric analysis of Western blots (relative to Figure 8A).

Dunnett’s Multiple Comparisons Test	Mean Diff.	95.00% CI of Diff.	Below Threshold?	Summary	Adjusted *p* Value
TXNIP					
CTRL vs. GH	2.499	2.387 to 2.611	Yes	****	<0.0001
CTRL vs. Ph	0.978	0.866 to 1.090	Yes	****	<0.0001
CTRL vs. GH/Ph	2.544	2.432 to 2.656	Yes	****	<0.0001
C-Myc					
CTRL vs. GH	−0.175	−0.287 to −0.063	Yes	**	0.0015
CTRL vs. Ph	−0.249	−0.361 to −0.137	Yes	****	<0.0001
CTRL vs. GH/Ph	0.882	0.770 to 0.994	Yes	****	<0.0001
p-AMPK					
CTRL vs. GH	−0.410	−0.522 to −0.298	Yes	****	<0.0001
CTRL vs. Ph	−0.140	−0.252 to −0.028	Yes	*	0.0110
CTRL vs. GH/Ph	−1.392	−1.504 to −1.280	Yes	****	<0.0001
p-AKT					
CTRL vs. GH	−0.428	−0.540 to −0.316	Yes	****	<0.0001
CTRL vs. Ph	−0.273	−0.385 to −0.161	Yes	****	<0.0001
CTRL vs. GH/Ph	0.441	0.329 to 0.553	Yes	****	<0.0001

**Table 2:** Densitometric analysis of western blots (relative to Figure 8B).

Dunnett’s Multiple Comparisons Test	Mean Diff.	95.00% CI of Diff.	Below Threshold?	Summary	Adjusted *p* Value
TXNIP					
CTRL vs. Ph	0.789	0.705 to 0.873	Yes	****	<0.0001
CTRL vs. 2DG	−0.590	−0.674 to −0.506	Yes	****	<0.0001
CTRL vs. 2DG/Ph	0.825	0.741 to 0.909	Yes	****	<0.0001
C-Myc					
CTRL vs. Ph	0.074	−0.009 to 0.158	No	ns	0.0918
CTRL vs. 2DG	0.252	0.168 to 0.336	Yes	****	<0.0001
CTRL vs. 2DG/Ph	0.523	0.439 to 0.606	Yes	****	<0.0001
p-AMPK					
CTRL vs. Ph	−0.165	−0.249 to −0.081	Yes	****	<0.0001
CTRL vs. 2DG	−0.241	−0.325 to −0.157	Yes	****	<0.0001
CTRL vs. 2DG/Ph	−1.232	−1.316 to −1.148	Yes	****	<0.0001
p-AKT					
CTRL vs. Ph	−0.120	−0.204 to −0.036	Yes	**	0.0035
CTRL vs. 2DG	−0.241	−0.325 to −0.157	Yes	****	<0.0001
CTRL vs. 2DG/Ph	0.078	−0.005 to 0.162	No	ns	0.0714

**Table 3:** Densitometric analysis of western blots (relative to Figure 8C).

Dunnett’s Multiple Comparisons Test	Mean Diff.	95.00% CI of Diff.	Below Threshold?	Summary	Adjusted *p* Value
p-JNK					
CTRL vs. Ph	0.125	−0.043 to 0.293	No	ns	0.2098
CTRL vs. GH	−0.299	−0.467 to −0.130	Yes	***	0.0001
CTRL vs. GH/Ph	−6.197	−6.365 to −6.028	Yes	****	<0.0001
CTRL vs. 2DG	−0.118	−0.287 to 0.050	No	ns	0.2530
CTRL vs. 2DG/Ph	0.084	−0.083 to 0.253	No	ns	0.5603
p-ERK					
CTRL vs. Ph	0.594	0.425 to 0.762	Yes	****	<0.0001
CTRL vs. GH	−0.262	−0.430 to −0.093	Yes	***	0.0009
CTRL vs. GH/Ph	−0.714	−0.883 to −0.546	Yes	****	<0.0001
CTRL vs. 2DG	0.001	−0.166 to 0.170	No	ns	>0.9999
CTRL vs. 2DG/Ph	0.553	0.385 to 0.722	Yes	****	<0.0001
p-p38					
CTRL vs. Ph	0.051	−0.116 to 0.220	No	ns	0.8885
CTRL vs. GH	−0.410	−0.579 to −0.241	Yes	****	<0.0001
CTRL vs. GH/Ph	−1.107	−1.276 to −0.938	Yes	****	<0.0001
CTRL vs. 2DG	−0.498	−0.667 to −0.330	Yes	****	<0.0001

## Data Availability

The datasets used and/or analyzed during the current study are available from the corresponding author upon reasonable request.

## Supplementary Materials

The following supporting information can be downloaded at: https://www.mdpi.com/article/10.3390/ijms24097741/s1.
