# Supplementary material for: The Oligostilbene Gnetin H Is a Novel Glycolysis Inhibitor That Regulates Thioredoxin Interacting Protein Expression and Synergizes with OXPHOS Inhibitor in Cancer Cells
Source: Int J Mol Sci. 2023 Apr 23;24(9):7741. doi: 10.3390/ijms24097741 (PMC10178141; doi:10.3390/ijms24097741)
Supplement: Supplementary file 1 [file ijms-24-07741-s001.zip › ijms-2252267-Figures S1 and S2 & Table S3-resub.pdf]

Suppl. Figure S1

A

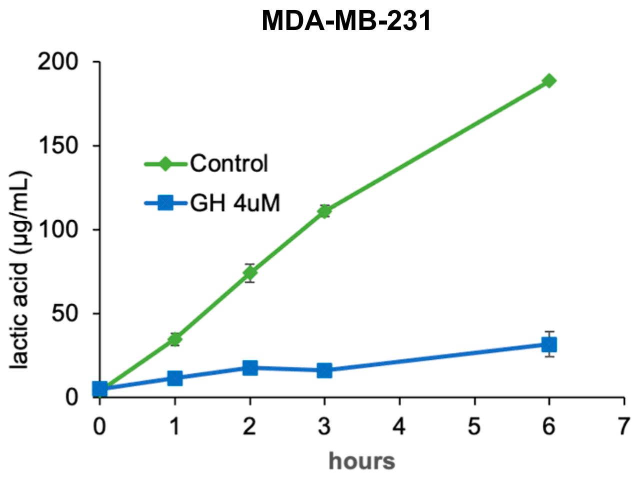

B

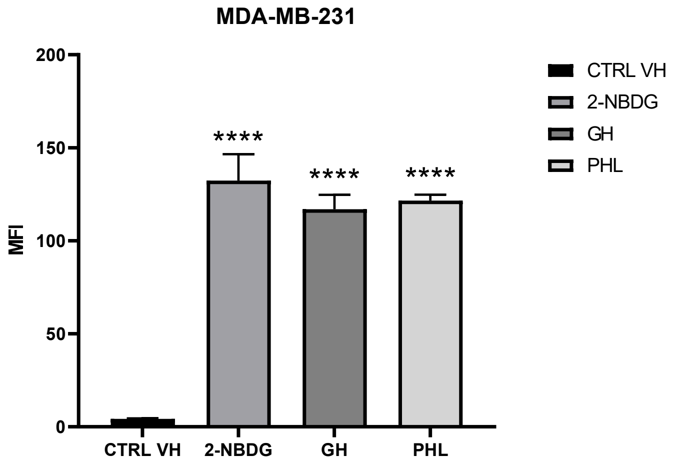

Supplementary Figure S1. MDA-MB-231 intracellular and extracellular lactic acid production and measurement of glucose transport. Panel A) MDA-MB-231 cells were treated with 4µM GH and the level of extracellular lactate was measured by sampling culture medium at 0, 1, 2, 3, and 6 hours. **Panel B)** Flow cytometric analysis of fluorescent glucose analog 2-NBDG following treatment with 4µM GH or phloretin, a glucose transporter inhibitor (One-Way Anova, Dunnet's post test, ****p<0.0001)

Suppl. Figure S2

A

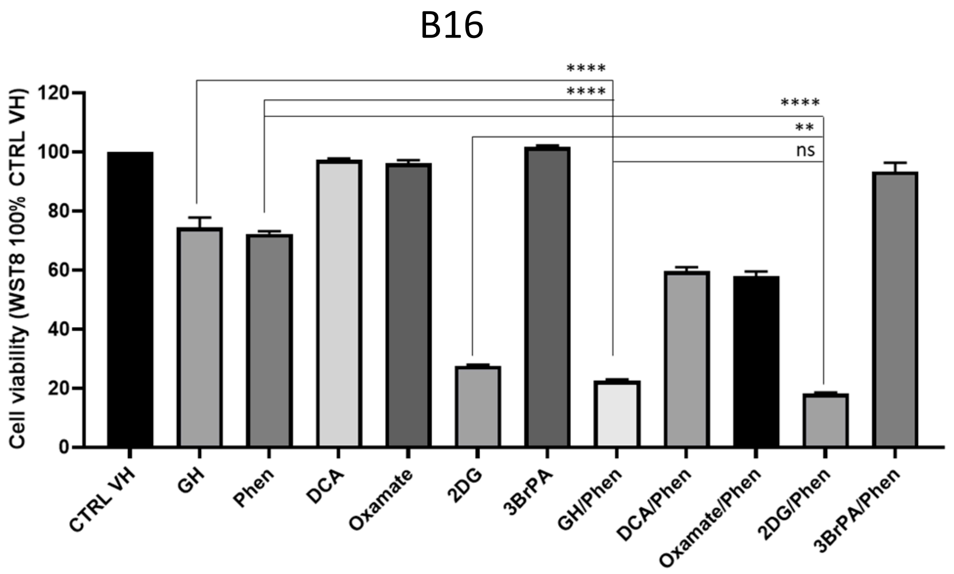

B

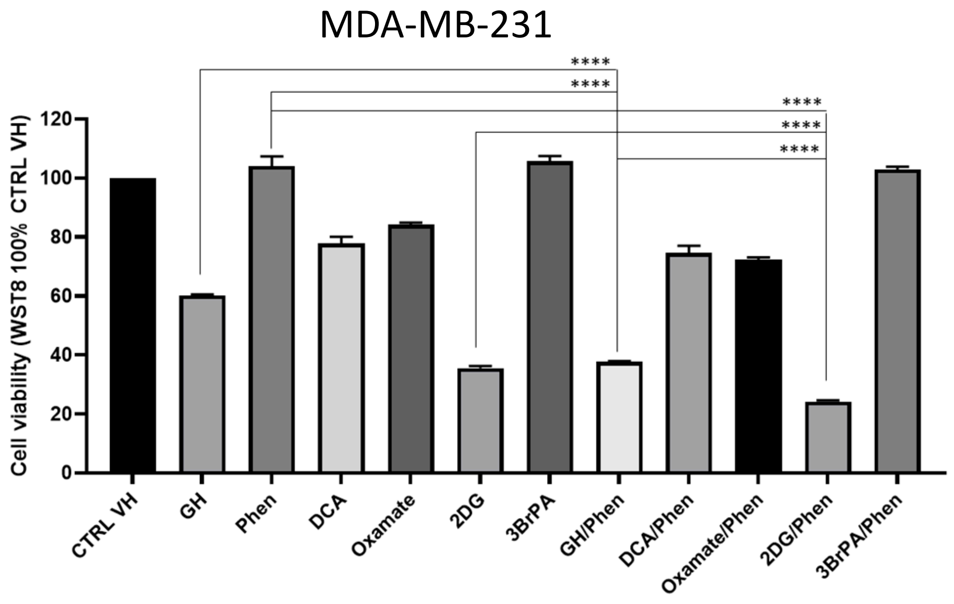

Supplementary Figure S2. Viability of B16 and MDA-MB231 cells following treatment with GH or other known glycolysis inhibitors in combination or not with mitochondrial complex-I inhibitor Phenformin.

Panel **A)** B16 or Panel **B)** MDA-MB-231 cells were treated for 48 hours with either 4µM GH or 25mM dichloroacetate (DCA), 25mM Oxamate, 25mM 2-Deoxy-D-glucose (2DG), 25µM 3- bromopyruvate (3BrPA), alone or in combination with 100µM Phenformin.

Profile of selected Metformin-Responding genes

Gene	Functional Roles[a]	Metformin[b]	Phen	2DG	GH	2DG + Phen	GH + Phen
ARRDC3	Protein ubiquitination, GPCR signaling pathway	Up	NC	NC	NC	+2.3	NC
CHAC1	Unfolded protein response and ER stress	Up	NC	+67.1	NC	NC	+13.1
DDIT3	Unfolded protein response and ER stress	Up	NC	+5.8	+2.5	+2.7	+3.8
DNAJB9	Unfolded protein response and ER stress	Up	NC	NC	+2.5	NC	+2.3
EGR1	Glucose and insulin response, and Wnt signaling	Up	NC	NC	NC	+4.7	NC
GDF15	SMAD, MAPK, and growth hormone signal transduction	Up	NC	+6.3	NC	+16.5	+5.3
JUN	SMAD and cAMP signal transduction, ER stress	Up	NC	NC	NC	+3.0	+2.0
ARRDC4	Glucose uptake, protein ubiquitination	Down	NC	+3.2	-5.8	+2.5	-5.1
TXNIP	Glucose uptake, cell cycle regulation	Down	-2.5	+2.2	-87.9	+2.1	-34.7

Up or +, Upregulated
Down or -, Downregulated
NC, No Change
Values represent fold-change with respect to solvent control
[a]Functional roles most relevant to the present study are listed
[b]Metformin transcriptional responses are based on Schulten et al. [46]
